# Modification of an observational tool to evaluate how community health workers engage low-income latino parents in parent–child interaction therapy

**DOI:** 10.3389/frcha.2026.1856265

**Published:** 2026-09-16

**Authors:** Hanan N. Salem, Francesca Rodriguez, Juliana Ison, Dainelys Garcia, Jason F. Jent, Eileen M. Davis, Betty Alonso, W. Andrew Rothenberg, Natalie Espinosa, Camille Perez, Yanet M. Rivera, Lilian M. Orbano-Flores, Carla Rincon, Miya L. Barnett

**Affiliations:** 1 Department of Counseling, Clinical, & School Psychology, University of California, Santa Barbara, CA, United States; 2 Mailman Center for Child Development, University of Miami, Miami, FL, United States; 3 ConnectFamilias, Miami, FL, United States; 4 Center for Child and Family Policy, Duke University, Durham, NC, United States

**Keywords:** behavior observation, community health worker (CHW), engagement, Latino, parent–child interaction therapy

## Abstract

**Background:**

Caregiver engagement in behavioral parent training (BPT) interventions can have a significant impact on treatment outcomes and retention. Yet, low-income families of color often experience low rates of engagement in treatment due to structural and sociocultural barriers. BPT models partnering with community health workers (CHWs) have shown promise in addressing these barriers and increasing treatment engagement. The current study examines the engagement strategies utilized by CHWs when providing support to families receiving Parent–Child Interaction Therapy (PCIT + CHW) by identifying common CHW engagement strategies utilized in treatment (e.g., active listening, psychoeducation) and how they influence components of engagement (e.g., alliance, attendance).

**Methods:**

Among families receiving a telehealth delivered PCIT + CHW model (*n* = 26), two recorded sessions (i.e., first session and a second randomly selected session throughout treatment) per family were coded for engagement strategy delivery. Families included primarily Latino children (96%) and their primary caregivers. Data about caregiver perceived therapeutic alliance, treatment attendance, homework completion, and PCIT skill acquisition were collected.

**Results:**

Findings suggest that, on average, CHWs utilized total strategies (i.e., sum of all strategies) and alliance-based strategies (i.e., active listening, conveying partnership, and positive regard) to a moderate extent. Alliance strategies delivered during the first session were associated with caregiver-perceived therapeutic alliance, caregiver-perceived effectiveness of the tasks guiding treatment, and caregiver homework completion in the first phase of treatment.

**Conclusion:**

Findings indicate CHWs use a range of engagement strategies, and early use of alliance-based strategies influence multiple components of engagement. Implications for training and involving CHWs in children's mental health services are discussed.

## 1 Introduction

Although behavioral parent training (BPT) programs have strong scientific evidence that support their use in promoting behavioral and emotional wellness among children and adolescents (1–4), over half of eligible families do not receive these services (5), with low-income families of color being most heavily impacted by common service engagement barriers (6–12). Barriers to treatment engagement endorsed by these underserved families include logistical barriers (e.g., transportation, health insurance, linguistically and culturally appropriate providers) and perceptual barriers (e.g., perceived need, stigma), which intensify mental health disparities and related long-term consequences these families experience (13, 14). Low treatment engagement not only exacerbates existing mental health disparities for families of color (6), but also affects provider confidence, burnout, and subsequent turnover (12, 15), further burdening mental health systems (16, 17). Given that improved treatment engagement can significantly enhance mental health care across individuals and systems (18–20), improving treatment engagement among underserved families can have a significant public health impact.

Engagement is often conceptualized as multidimensional, as it involves the interaction of cognitive (e.g., readiness for change, attitudes to treatment), behavioral (e.g., in-session participation, homework completion), and social (e.g., therapeutic alliance) dimensions (18, 21–23). REACH, an engagement framework that has been evaluated to demonstrate generalizability across youth and caregivers of color (24), suggests engagement is comprised of the following five dimensions: relationship (e.g., alliance), expectancy (e.g., perspectives about treatment and its benefits), attendance (e.g., attending sessions), clarity (e.g., understanding the treatment and the roles of those participating in treatment), and homework (e.g., participation in session and at home (22, 25). The present study operationalizes caregiver engagement in treatment by evaluating the components of the REACH framework that capture the cognitive, behavioral, and social dimensions of engagement.

While it is important to consider and address the system-level components that can act as a barrier or facilitator to treatment engagement, research also suggests provider strategies utilized in treatment can influence engagement (23, 26, 27). Accordingly, engagement literature suggests that the provider strategies found to promote engagement outcomes include rapport building (e.g., emphasizing partnership, active listening), assessing for needs and strengths, assessing and addressing participation barriers, collaborative problem-solving, homework assignment, goal setting, treatment expectations and psychoeducation (e.g., information about treatment and behaviors (20–22, 27, 28). These elements frequently occur alongside one another and in conjunction with other elements.

Despite research supporting the use of these strategies to increase engagement in treatment, families of color continue to experience lower rates of engagement compared to white families, with notable differences in caregivers’ willingness to share their perspectives on treatment (27, 29). This suggests there may be cultural differences that need to be considered when engaging families of color (30), with some studies suggesting that client-provider match and/or understanding across various factors, such as race, culture, and socioeconomic status, can significantly improve treatment engagement outcomes (20).

Research suggests that leveraging community resources, such as partnering with community health workers (CHWs), has shown promise in increasing access to mental health services and reducing engagement disparities within these underserved communities (31–35). CHWs are lay members within a community that serve as a link between their community and health providers to address health service barriers and disparities (36). CHWs leverage their shared proximity (e.g., shared experiences, culture, language) to engage individuals and families in treatment and address inequities impeding access and engagement (33). Indeed, CHWs are uniquely equipped to address common barriers low-income families of color experience because they are embedded within these same communities and, therefore, have the familiarity and knowledge to develop trusting relationships within the community and advocate for community members. They also address demand and supply challenges within the mental health system by increasing the supply of culturally appropriate providers when trained to provide mental health services and interventions (33, 37).

Qualitative studies have been conducted to further explore how CHWs engage individuals and, in particular, caregivers in treatment. Gustafson and colleagues (38), for example, interviewed CHWs embedded within a school-based setting serving predominantly Latino and Black students to understand their use of engagement strategies with caregivers of school-age students. The CHWs in this study delivered skill-building support for students and parenting support as well as additional case management, family engagement and school-family liaison services. They found that CHWs endorsed using rapport-building strategies, such as supportive listening, leveraging shared experiences, acknowledging the caregiver's efforts, emphasizing confidentiality, and leveraging relationships within the school. Moreover, strategies such as increasing flexibility of services, consistency of outreach, advocacy, incentivizing caregiver participation, and meeting the family where they were promoted direct service utilization. Similarly, Barnett et al. (39) interviewed community-embedded and agency-embedded CHWs to understand engagement strategies used. Community-embedded CHWs primarily connected families to services and sometimes delivered prevention parenting programs, whereas agency-embedded CHWs provided in-home mental health support and helped caregivers navigate other systems. Across CHWs, they found that rapport and trust building (e.g., listening, leveraging shared identities, self-disclosures, meeting them where they are), empowerment (e.g., advocacy and providing information), and increasing access (e.g., providing resources and bridging services) were strategies CHWs found to be important in their roles supporting families through treatment.

Parent–Child Interaction Therapy (PCIT) is an evidence-based BPT intervention developed to address child behavior problems (40). Through this intervention caregivers are first taught skills (i.e., increasing “do skills,” such as praises and desisting from leading “avoid behaviors” like commands) to promote a warm and secure caregiver-child relationship. They are then provided with discipline strategies that are consistent and predictable to promote limit setting. The intervention involves live coaching, in which the provider guides caregivers *in-vivo* through use of the skills within each phase of treatment until goals are met. There is strong evidence suggesting that PCIT is effective at increasing positive parenting skills and decreasing challenging child behaviors within low-income children and families of color that complete treatment (41). The coaching component of PCIT also makes it uniquely well-suited to be delivered via telehealth (42, 43).

The PCIT plus community health worker (PCIT + CHW) model partners with CHWs to provide culturally responsive support to underserved families undergoing PCIT. This auxiliary support CHW model aims to increase reach and treatment engagement within low-income, racially and/or ethnically diverse families by leveraging CHWs to identify families with unmet needs within these underserved communities and introduce PCIT to them. In this intervention model, in additional to traditional PCIT sessions with a therapist, families also work with a CHW who provides support during treatment by addressing barriers to treatment attendance and homework completion, and assisting with caregiver skill acquisition (i.e., do skills) and desistence (i.e., avoid skills) in the home. CHWs work closely with the family's PCIT therapist to determine additional needs as well as progress with skills acquisition and desistence. For additional information regarding the PCIT + CHW model, see Davis et al. (44) and Garcia et al. (45).

An open trial evaluating the PCIT + CHW model suggests this model is effective in reaching communities not served within typical academic medical settings (e.g., university-based clinics) and showed positive outcomes with respect to treatment attendance, skill acquisition and desistence, and caregiver stress (44). Furthermore, a randomized controlled trial (RCT) comparing standard PCIT to the PCIT + CHW model within families recruited from traditionally underserved communities demonstrated comparable engagement outcomes and better clinical outcomes, with significantly improved child externalizing symptoms and child compliance among families receiving the PCIT + CHW model compared to standard PCIT (45). These preliminary findings are encouraging, but little is known about how the CHW support influences caregiver engagement in treatment. The current study aims to understand how varying engagement strategies and behaviors observed in the CHW sessions influence child and caregiver outcomes.

Given the evidence demonstrating that low-income families of color often encounter barriers limiting their engagement in treatment (8, 11), which can significantly impact treatment outcomes (10, 46), and CHW treatment models have been shown to reduce disparities in care (44, 45), it is imperative to explore the ways in which CHW engagement strategies influence treatment engagement among traditionally underserved families. The proposed study aims to directly observe engagement strategies utilized by CHWs by coding video recorded PCIT + CHW sessions (i.e., first session and a randomly selected second session across the course of treatment) across low-income, predominantly Latino families. Specific study aims include: (1) identifying the engagement strategies utilized by CHWs in sessions with the families and (2) exploring how these strategies and behaviors observed within the CHW sessions influence engagement in treatment (i.e., relationship, expectancy, attendance, clarity, and homework). Given the limited research examining specific CHW engagement strategies within PCIT, the present study was exploratory and did not specify *a priori* hypotheses. The observational data collected from CHW sessions and findings from this study can be critical to further understanding and improving treatment engagement and outcomes for underserved families across various settings and treatment models.

## 2 Methods

### 2.1 Participants

Participants were drawn from a pilot study being conducted at the University of Miami, in which families opted in to receiving the PCIT + CHW model via telehealth during the COVID-19 pandemic. See Davis et al. (44) for additional information regarding recruitment of families in the original pilot study. All families recruited for the pilot study with at least two recorded sessions were included in the present study. Participants in the current study included 26 children between the ages of 2 and 8 (*M* = 4.78; SD = 1.72) and their primary caregivers (*M* age = 36.25; SD = 8.17). Twenty-four of the caregivers identified as the child's biological mother, one caregiver identified as the child's biological father, and one caregiver identified as the child's adoptive mother. Participants for the pilot study were recruited from three PCIT clinics embedded within community agencies that typically serve predominantly low-income Latino and Black communities. Fifty percent of all families reported household incomes that were below the federal poverty line. All caregivers participating in the current study and 96% of the children were identified as being Latino and/or Hispanic. Twenty-three of the participating families (88.5%) received CHW sessions in Spanish. See Table 1 for additional child and caregiver characteristics.

**Table 1:** Participant characteristics.

Participant characteristics	Frequency (%)	M (SD)
Child		
Age (in years)		4.78 (1.72)
Sex (Female)	13 (50%)	
Ethnicity (Latino)	25 (96.15%)	
Race		
White	18 (69.23%)	
Black/African American	1 (3.85%)	
More than one race	2 (7.69%)	
Race not listed	5 (19.23%)	
Caregiver		
Age (in years)		36.25 (8.17)
Sex (Female)	24 (92.31%)	
Ethnicity (Latino)	26 (100%)	
Race		
White	19 (73.08%)	
Black/African American	-	
More than one race	-	
Race not listed	7 (26.92%)	
Highest Level of Education		
High School/GED or Less	4 (15.38%)	
Some College/Associate Degree	5 (19.23%)	
Bachelor's Degree	13 (50%)	
Graduate Degree	4 (15.38%)	
Proficient in English (Yes)	15 (57.69%)	
Marital Status (Married or Domestic Partnership)	15 (57.69%)	

### 2.2 Procedures

#### 2.2.1 PCIT + CHW intervention

The present study included three CHWs who were trained in the PCIT + CHW model. The CHWs in the current study did not have formal graduate-level mental health training. Two of the three CHWs reported bachelor's degree and one CHW reported high school as their highest level of education. All three CHWs completed a structured PCIT training focused on family engagement, parenting skills, behavioral observation, feedback, home-practice support, and addressing barriers to treatment. These CHWs assisted with participant recruitment and provided telehealth support to the families opting to receive the PCIT + CHW treatment model. During these sessions, CHWs assisted families by supporting their learning of parenting principles in a culturally responsive manner, aiding them in acquiring parenting skills through observing and coaching parents during parent–child interactions in the home, and addressing structural barriers to accessing and benefiting from services (e.g., access to technology, food and housing challenges, immigration needs). CHWs participated in trainings in which they learned skills to effectively support families in recruitment, engagement, and skill acquisition. See Davis et al. (44) and Garcia et al. (45) for additional information. During the transition to virtual services due to the COVID-19 pandemic, all therapists and staff supporting families through services received a telehealth training, in which they learned about virtual service delivery, with a focus on the delivery of I-PCIT as well as common challenges that arise while delivering services virtually (47). Participants receiving PCIT + CHW received up to 18 weeks of weekly internet-delivered PCIT (I-PCIT; *M* = 11.69; SD = 2.83) with a PCIT therapist and approximately biweekly telehealth sessions with a CHW (*M* = 7.35; SD = 2.76, *range* = 2–13). CHW sessions occurred an average of 1.86 weeks apart (SD = 0.56 weeks) and were video recorded and stored on a secure cloud-based drive.

#### 2.2.2 Assessment

All study procedures were approved by the University of Miami Institution Review Board (IRB). Caregivers completed questionnaires at four times points: (1) before treatment began, (2) mid-treatment, (3) post-treatment, and (4) one-month follow-up. All surveys were collected and stored online via the research electronic data capture (REDCap) system (48). After each CHW session, the CHW uploaded the recorded session and completed a progress note to document the session. Families with at least two recorded sessions were selected to participate in the present study. The first session and a second randomly selected session were coded across all participating families. The timing of the second observation varied across participants, occurring at an average of 4.42 CHW sessions (SD = 2.27; *range* = 2–10) into their course of CHW support.

### 2.3 Measures

#### 2.3.1 Participant characteristics

Caregivers were asked to provide demographic information, including age, gender, race, and ethnicity for both the child and primary caregiver in treatment.

#### 2.3.2 Engagement strategies utilized by CHWs

##### 2.3.2.1 Therapist alliance, collaboration, and empowerment strategies (ACEs) observational coding system

The Therapist ACEs Observational Coding System is a 12-item observational tool used to evaluate the frequency and intensity of provider engagement strategies on four domains: (1) alliance, (2) collaboration, (3) empowerment, and (4) psychoeducation (see Table 2 for examples of codes within each domain). Coders provide a global extensiveness rating for each engagement strategy on a seven-point Likert scale (0–6). Extensiveness reflects two dimensions: the presence/frequency (i.e., whether the strategy was implemented at all and the amount of times it occurred) and thoroughness (i.e., the quality and completeness with which the strategy is implemented) of the strategy. Higher scores indicating greater extensiveness. The ACEs Observational Coding System has demonstrated acceptable internal consistency (*α* = 0.60–0.72) and fair to excellent interrater reliability (ICCs = 0.44–0.80) across samples of racially and/or ethnically diverse families and community-based mental health therapists (27, 49).

**Table 2:** Item level base rate and intraclass correlation coefficient (ICC) analyses.

Item (*n* = 52 PCIT + CHW sessions coded)	Base rate	ICC
Alliance		
CHW actively listened to caregiver's perspectives	76.9%	0.599
CHW conveyed caregiver -CHW partnership in child's therapy	94.2%	0.592
CHW communicated positive regard to caregiver	75.0%	0.545
CHW leveraged shared characteristics, background, or lived experiences to build trust	13.5%	0.788
Collaboration		
CHW offered suggestions about therapeutic tasks	96.2%	0.203
CHW sought out caregiver input	96.2%	0.42
CHW incorporated caregiver's input into therapeutic tasks	21.2%	0.517
CHW attended to caregiver's actions taken outside of session	67.3%	0.452
CHW met the caregiver where they were at	61.5%	0.172
Empowerment		
CHW recognized caregiver strengths and effort to participate in therapy	84.6%	0.915
CHW addressed barriers to caregiver participation	15.4%	0.6
CHW modeled advocacy to the caregiver when navigating various systems	5.8%	-
Psychoeducation		
CHW provided psychoeducation about child problems to caregiver	36.5%	0.62
CHW provided psychoeducation about child services to caregiver	98.1%	0.06

###### 2.3.2.1.1 Modification of the ACES

The Therapist ACEs was modified to remove non-relevant items given the nature of PCIT (i.e., *therapist involved caregiver in therapeutic activities*) and include common engagement strategies as identified by CHWs in previous studies as unique ways they engage caregivers (39): *CHW leveraged shared characteristics, background, or lived experiences to build trust*, *CHW met the caregiver where they were at*, and *CHW modeled advocacy to the caregiver when navigating various systems*. In the modified version of the observational coding system, coders provided a global extensiveness rating consisting of both the frequency/consistency and extensiveness of each engagement strategy on a 4-point Likert scale (0–3; original scale was on a 7-point Likert scale from 0 to 6) with higher scores indicating more frequent and thorough use of the strategy. Given that the original scale merged values across descriptors (e.g., a score of 5 or 6 indicated consistent and thorough use of a strategy), scale values were merged to clearly differentiate descriptors and, thus, increase rater agreement (50). In general, a rating of 0 indicated that the strategy was not used, whereas ratings of 1–3 reflected increasing levels of frequency/consistency and thoroughness. Anchors were operationalized separately for each strategy. For example, active listening was defined as: CHW actively listened to parent's perspectives by a) repeating or paraphrasing parent's perspectives, OR b) summarizing multiple points the parent has made, OR c) reflecting back parent's underlying feelings. A rating of 0 indicated no attempts to actively listen; 1 indicated few and/or limitedly thorough attempts; 2 indicated several and/or moderately thorough attempts; and 3 indicated consistent, recurrent and/or highly thorough attempts. Thus, ratings reflected a global assessment of both how consistently and how thoroughly a strategy was implemented rather than frequency alone.

###### 2.3.2.1.2 Coder reliability training

The coding team consisted of two graduate-level students and one post-baccalaureate research assistant. Coders attended a didactic training led by the first author and completed three rounds of practice coding in which they coded a total of 12 practice recordings. Training was completed in a total of 8 weeks, with bi-weekly meetings to clarify code discrepancies. Coders began independently coding videos after the third round of practice, in which they achieved acceptable levels of agreement with the gold-standard codes (an average of 80% agreement within ±1 point on Likert scale when a strategy is observed across four recordings).

###### 2.3.2.1.3 Independent coding procedures

Coders fully watched each recorded CHW session and noted observed CHW engagement strategies. Coders then assigned a global extensiveness rating from 0 (*not observed at all*) to 3 (*observed to a great extent*) for each engagement strategy. To test for interrater reliability, 15 CHW sessions (29%) were randomly selected to be double coded. One-way random effects, absolute agreement, multiple raters/measurements intraclass correlation coefficients (ICCs) were calculated for each code to determine interrater reliability (51). Final codes were included in analyses if they had a base rate of at least 15% and demonstrated acceptable interrater reliability (52).

###### 2.3.2.1.4 Final codes

Base rates and ICCs for each engagement strategy are presented in Table 2. Due to low reported rates, the following two codes were not included in analyses: *CHW leveraged shared characteristics, background, or lived experiences to build trust* and *CHW modeled advocacy to the caregiver when navigating various systems*. Of the remaining items, the following three items demonstrated low ICCs: *CHW offered suggestions about therapeutic tasks*, *CHW met the caregiver where they were at*, and *CHW provided psychoeducation about child services to caregiver.* The researchers further examined these codes and found that two of the three codes (*CHW offered suggestions about therapeutic tasks* and *CHW provided psychoeducation about child services to caregiver*) exhibited high levels of agreement (at least 80% agreement within ±1 point when a strategy is observed) yet demonstrated low variability due to consistently high reporting of these items. Previous research suggests ICC analyses are sensitive to variability among samples and lack of variability can distort resulting coefficients and interpretations (51, 53). Given these findings and high levels of rater agreement among the two codes, they were both included in the final analyses, yielding a total of 11 final codes.

McDonald's omega (*ω*) (54); was calculated for each composite and the total use of engagement strategies. Only the alliance composite (*ω* = 0.66) and the total score (*ω* = 0.73) demonstrated acceptable internal consistency. Therefore, the alliance composite score (i.e., sum across the remaining three alliance codes) and the total engagement strategy score (i.e., sum across all 11 final codes) were included in analyses in addition to the 11 individual final codes.

#### 2.3.3 Measures of caregiver engagement

Measures were completed to capture every facet of the REACH framework. Notably, treatment completion was not included in analyses because all but three participants (88.5%) completed treatment.

##### 2.3.3.1 Relationship

The Working Alliance Inventory—Short Revised [WAI-SR (55)]; is a 12-item self-report questionnaire used to assess three domains of therapeutic alliance: (1) agreement of goals in therapy, (2) agreement of the tasks of therapy, and (3) the bond between the provider and client. For the current study, caregivers were asked to think about their experience with their CHW and rate each item on a five-point Likert scale from 1 (*Seldom*) to 5 (*Always*), with higher scores suggesting greater alliance. The WAI-SR has demonstrated good reliability across the scales and has been validated in Spanish (*α* = 0.85–0.91 (55, 56). The bond subscale and total score at post-treatment evaluated the therapeutic relationship between the CHW and caregiver. Examples of items include *[my provider] and I respect each other* and *I feel [my provider] cares about me even when I do things that he/she does not approve of*.

##### 2.3.3.2 Expectancy

The task subscale (i.e., agreement of the tasks of therapy) of the WAI-SR was used to assess the caregiver's perceptions regarding treatment. Examples of items on this subscale include *I feel that the things I do in therapy will help me to accomplish the changes that I want* and *I believe the way we are working with my problem is correct*.

##### 2.3.3.3 Attendance

Treatment attendance was measured by dividing the total number of coach sessions caregivers completed by the total number of weeks in which they were in treatment after intake, excluding weeks during which a teach session was conducted.

##### 2.3.3.4 Clarity

The Dyadic Parent–Child Interaction Coding System, Fourth Edition [DPICS-IV (57)]; is a behavior observation tool used to measure the acquisition of positive parenting skills and desistence of negative parenting skills. During the five-minute Child-Led Play (CLP) condition, caregivers are instructed to follow their child's lead in play while the observer records the number of positive or do skills (i.e., behavior descriptions, reflections, and labeled praises) as well as the number of avoid behaviors (i.e., questions, commands, criticisms). A caregiver meets criteria goals when 10 or more of each do skill and 3 or fewer total avoid behaviors is recorded in a given five-minute parent–child interaction. PCIT therapists trained to 80% coding reliability coded the do and avoid skills during treatment sessions. The do and avoid skills from intake to post-treatment were used to measure changes in skill acquisition and desistence before and after treatment. These changes in skills were used to assess the caregiver's understanding of treatment.

##### 2.3.3.5 Homework

Homework completion rates were calculated separately across the two phases of treatment [i.e., Child-Directed Interaction [CDI] and Parent-Directed Interaction [PDI]]. Homework completion was calculated at each session by dividing the number of days during which homework was completed by the total number of possible days homework could have been completed between sessions. The overall rates across the phases of treatment were then averaged out to yield the following three rates of homework completion: (1) CDI homework completed during the CDI phase, (2) CDI homework completed during the PDI phase, and (3) PDI homework completed during the PDI phase.

### 2.4 Analytic plan

All engagement strategies, the alliance composite, and the total score were analyzed across the two time points (i.e., the first session and a randomly selected second session across the course of the intervention). In addition, the average for each item was taken across the two time points to yield an average item rating as well as an average for each composite score and individual engagement strategies. Given that engagement is dynamic and changes over time (23, 25), the averaged variable was included to provide a more stable and reliable estimate of engagement strategies over time by reducing session-specific variability and random measurement error (58). Descriptive statistics were conducted to determine the use of the engagement strategies across time points. Ordinary least squares (OLS) simple linear regression models were then run to examine the relationship between the independent variable, engagement strategies utilized by CHWs in session, and the dependent variable, treatment engagement components (i.e., relationship, expectancy, attendance, clarity, and homework). Given the exploratory nature of the analyses, corrections for multiple comparisons were not applied. Findings were interpreted as preliminary and hypothesis-generating.

## 3 Results

### 3.1 Use of engagement strategies

Means and standard deviations for each engagement strategy, the alliance composite, and the total use of engagement strategies are presented in Table 3. On average, CHWs utilized strategies to a moderate extent (*M* = 13.94) and alliance strategies were low to moderate (*M* = 3.85) across sessions. CHWs utilized the following strategies with little consistency and/or thoroughness (*M* = 0.25–0.83): *addressing barriers*, *incorporating caregiver's input*, *providing psychoeducation about child problems*, and *attending to the caregiver's actions outside of session*. Overall, rater-observed strategies utilized to a great extent included the CHW *recognizing the caregiver's strengths and effort to participate in therapy* (e.g., praising the caregiver's skill use and participation; *M* = 2.10) and the CHW's *psychoeducation of services* (*M* = 2.65).

**Table 3:** Engagement strategies and composite scores.

	Time 1 (session 1)		Time 2 (randomly selected)		Average across time 1 and 2	
	*M*	SD	*M*	SD	*M*	SD
Engagement strategy (item max = 3)						
Alliance						
CHW actively listened to caregiver's perspectives	1.12	0.82	1.15	0.93	1.13	0.71
CHW conveyed caregiver-CHW partnership in child's therapy	1.46	0.71	1.42	0.64	1.44	0.45
CHW communicated positive regard to caregiver	1.50	0.95	1.04	1.08	1.27	0.70
Collaboration						
CHW offered suggestions about therapeutic tasks	1.81	0.63	1.69	0.74	1.75	0.45
CHW sought out caregiver input	1.50	0.76	1.69	0.74	1.60	0.55
CHW incorporated caregiver's input into therapeutic tasks	0.31	0.62	0.35	0.80	0.33	0.55
CHW attended to caregiver's actions taken outside of session	0.88	0.77	0.77	0.59	0.83	0.51
Empowerment						
CHW recognized caregiver strengths and effort to participate in therapy	2.04	0.53	2.15	0.73	2.10	0.47
CHW addressed barriers to caregiver participation	0.19	0.57	0.31	0.74	0.25	0.43
Psychoeducation						
CHW provided psychoeducation about child problems to caregiver	0.65	0.94	0.54	0.86	0.60	0.72
CHW provided psychoeducation about child services to caregiver	2.77	0.43	2.54	0.58	2.65	0.34
Composite						
Alliance Composite (max = 12)	4.08	1.85	3.62	2.12	3.85	1.43
Total (max = 33)	14.23	3.64	13.65	4.66	13.94	3.03

### 3.2 Strategies predicting engagement outcomes

Tables 4–6 present the results from linear regression models at time 1, time 2, and the average across times 1 and 2, respectively, when examining alliance and total strategy use. The results are summarized according to the REACH engagement components. Table 7 provides a summary of the significant findings specific to the REACH engagement components.

**Table 4:** Engagement strategy predictors of REACH engagement components at time 1.

Engagement component: variable	*B*	*SE*	*R* ^2^	*p*
Relationship: WAI-SR Total Score				
Alliance Composite	1.78	0.75	0.29	0.03
Total Score	0.9	0.4	0.26	0.04
Relationship: WAI-SR Bond				
Alliance Composite	0.58	0.25	0.22	0.04
Total Score	0.28	0.14	0.23	0.06
Expectancy: WAI-SR Task				
Alliance Composite	0.61	0.27	0.26	0.04
Total Score	0.3	0.15	0.23	0.06
Attendance: Coach Show Rate				
Alliance Composite	1.96	2.14	0.03	0.37
Total Score	0.84	1.1	0.02	0.45
Clarity: Do Skills				
Alliance Composite	−0.29	1.19	0.12	0.81
Total Score	0.06	0.66	0.11	0.93
Clarity: Don't Skills				
Alliance Composite	−0.54	0.55	0.16	0.34
Total Score	−0.18	0.32	0.13	0.58
Homework: CDI in CDI				
Alliance Composite	3.18	1.77	0.12	0.09
Total Score	1.25	0.93	0.07	0.19
Homework: CDI in PDI				
Alliance Composite	0.39	2.36	0	0.87
Total Score	0.2	1.2	0	0.87
Homework: PDI in PDI				
Alliance Composite	1.95	3.04	0.02	0.53
Total Score	0.4	1.56	0	0.8

WAI-SR, Working Alliance Inventory—Short-form; CDI, child-directed interactions; PDI, parent-directed interactions.

**Table 5:** Engagement strategy predictors of REACH engagement components at time 2.

Engagement component: variable	*B*	*SE*	*R* ^2^	*p*
Relationship: WAI-SR Total Score				
Alliance Composite	−0.28	0.74	0.01	0.72
Total Score	−0.11	0.32	0.01	0.75
Relationship: WAI-SR Bond				
Alliance Composite	0.03	0.25	0.00	0.89
Total Score	0.02	0.11	0.00	0.83
Expectancy: WAI-SR Task				
Alliance Composite	−0.23	0.26	0.05	0.39
Total Score	−0.09	0.11	0.05	0.43
Attendance: Coach Show Rate				
Alliance Composite	1.37	1.89	0.02	0.47
Total Score	1.31	0.82	0.10	0.13
Clarity: Do Skills				
Alliance Composite	0.48	1.22	0.12	0.70
Total Score	0.14	0.59	0.12	0.82
Clarity: Don't Skills				
Alliance Composite	−0.24	0.50	0.13	0.63
Total Score	−0.12	0.22	0.13	0.61
Homework: CDI in CDI				
Alliance Composite	2.31	1.58	0.08	0.16
Total Score	0.58	0.74	0.03	0.44
Homework: CDI in PDI				
Alliance Composite	2.22	2.01	0.05	0.28
Total Score	−0.02	0.94	0.00	0.98
Homework: PDI in PDI				
Alliance Composite	0.83	1.87	0.02	0.66
Total Score	−1.69	1.17	0.08	0.16

WAI-SR, Working Alliance Inventory—Short-form; CDI, child-directed interactions; PDI, parent-directed interactions.

**Table 6:** Engagement strategy predictors of REACH engagement components across times 1 and 2.

Engagement component: variable	*B*	*SE*	*R* ^2^	*p*
Relationship: WAI-SR Total Score				
Alliance Composite	0.99	1.02	0.06	0.35
Total Score	0.37	0.48	0.04	0.45
Relationship: WAI-SR Bond				
Alliance Composite	0.45	0.33	0.05	0.20
Total Score	0.18	0.16	0.09	0.27
Expectancy: WAI-SR Task				
Alliance Composite	0.20	0.37	0.02	0.60
Total Score	0.06	0.18	0.01	0.75
Attendance: Coach Show Rate				
Alliance Composite	3.16	2.76	0.05	0.26
Total Score	2.16	1.26	0.11	0.10
Clarity: Do Skills				
Alliance Composite	0.17	1.70	0.11	0.92
Total Score	0.20	0.86	0.12	0.82
Clarity: Don't Skills				
Alliance Composite	−0.75	0.74	0.16	0.32
Total Score	−0.28	0.37	0.14	0.45
Homework: CDI in CDI				
Alliance Composite	5.23	2.21	0.19	0.03
Total Score	1.59	1.11	0.08	0.17
Homework: CDI in PDI				
Alliance Composite	2.78	3.01	0.03	0.37
Total Score	0.12	1.44	0.00	0.94
Homework: PDI in PDI				
Alliance Composite	−0.59	3.98	0.00	0.88
Total Score	−1.72	1.84	0.04	0.36

WAI-SR, Working Alliance Inventory—Short-form; CDI, child-directed interactions; PDI, parent-directed interactions.

**Table 7:** Summary of significant engagement strategy predictors of REACH engagement components.

Engagement component	Engagement strategy/composite	Time point	*B*
Relationship: WAI-SR Total Score	Alliance Composite	Time 1	1.78
Relationship: WAI-SR Total Score	Total Score	Time 1	0.90
Relationship: WAI-SR Total Score	*CHW communicated positive regard to caregiver*	Time 1	3.19
Relationship: WAI-SR Total Score	*CHW provided psychoeducation about child problems to caregiver*	Time 1	10.14
Relationship: WAI-SR Bond	Alliance Composite	Time 1	0.58
Relationship: WAI-SR Bond	*CHW provided psychoeducation about child problems to caregiver*	Time 1	3.71
Expectancy: WAI-SR Task	Alliance Composite	Time 1	0.61
Attendance: Coach Show Rate	*CHW provided psychoeducation about child problems to caregiver*	Time 1	8.77
Attendance: Coach Show Rate	*CHW provided psychoeducation about child problems to caregiver*	Average of T1 and T2	13.72
Clarity: Do Skills	CHW provided psychoeducation about child problems to caregiver	Average of T1 and T2	−8.54
Homework: CDI in CDI	Alliance Composite	Average of T1 and T2	5.23

WAI-SR, Working Alliance Inventory—Short-form; CDI, child-directed interactions.

#### 3.2.1 Relationship

To explore the association between the delivery of engagement strategies in session and the CHW-caregiver relationship, the strategies were regressed on the WAI-SR total and bond scores. Alliance strategy (*B* = 1.78, *p* = 0.03) and total strategy use (*B* = 0.90, *p* = 0.04) during the first CHW session were positively and significantly associated with the WAI-SR total score (i.e., caregiver positive perceptions of working alliance with their CHWs). When examining individual strategies at time 1, *CHW communicated positive regard to caregiver* (*B* = 3.19, *p* = 0.03) and *CHW provided psychoeducation about child problems to caregiver* (*B* = 10.14, *p* = 0.02) were associated with WAI-SR total score. Total alliance strategy use at time 1 (*B* = 0.58, *p* = 0.04) and *CHW provided psychoeducation about child problems to caregiver* (*B* = 3.71, *p* = 0.01) were also associated with the WAI-SR bond score. These findings highlight the utility of alliance strategies and psychoeducation in the first session with respect to CHW-caregiver relationship building. No other strategies at time 1, time 2, or across both times significantly predicted WAI-SR total or bond score.

#### 3.2.2 Expectancy

The influence of engagement strategy delivery on caregiver perceptions about the benefit and effectiveness of treatment was evaluated by regressing the strategy on the WAI-SR task score. Accordingly, total use of alliance strategies (*B* = 0.61, *p* = 0.04) was significantly associated with the WAI-SR task score. This, again, emphasizes first session alliance strategy use to build treatment expectations. No other strategies predicted WAI-SR task score across the various timepoints.

#### 3.2.3 Attendance

To evaluate the relationship between the utilization of engagement strategies in session and overall attendance, strategy use was regressed on the coach show rate (i.e., attendance rate across coach sessions). Linear regression models indicate that *CHW provided psychoeducation about child problems to caregiver* at time 1 (*B* = 8.77, *p* = 0.04) and when averaged across both times 1 and 2 (*B* = 13.72, *p* = 0.01) significantly influenced the attendance rate. In other words, psychoeducation, especially about behavioral concerns, was shown to be an important strategy that had an impact on overall attendance. No other strategies influenced attendance rates across time points.

#### 3.2.4 Clarity

Skill acquisition (i.e., do skills) and desistence (i.e., avoid behaviors) were evaluated to explore the relationship between strategy delivery and caregiver understanding of the skills. When strategies were regressed on total do skills after the completion of treatment (i.e., the difference between do skills from intake to post-treatment), average use of *psychoeducation about child problems to caregiver* across times 1 and 2 was negatively associated (*B* = −8.54, *p* = 0.03). No other strategies were found to be significant across timepoints when evaluating the influence of strategy use on total do and avoid skills after the completion of treatment.

#### 3.2.5 Homework

To evaluate the influence of strategy use on homework completion, three types of homework rates were observed: homework completion in the first phase of treatment (i.e., special time for relationship enhancement) and second phase of treatment (i.e., special time for continued relationship enhancement and separate practice of discipline skills). Overall use of the alliance strategies across timepoints (*B* = 5.23, *p* = 0.03) was associated with homework completion rates in the first phase of treatment. No other strategies across time points were found to influence homework completion rates during the first or second phase of treatment.

## 4 Discussion

One aim of the present study was to identify engagement strategies CHWs utilize while delivering PCIT + CHW sessions using a modified observational tool. Studies exploring engagement practices within CHW treatment models have primarily focused on collecting qualitative interviews from CHWs (38, 39). Moreover, the observer-rated data collection method provides a measure of engagement that is more objective than self-report instruments (59). A number of studies have utilized similar types of observational methods to measure engagement strategies and behaviors within families receiving various EBTs (12, 60–62), but, to the author's knowledge, there are no other studies utilizing observational tools to evaluate engagement in CHW models of behavioral and mental health care. CHWs are tasked with addressing engagement disparities for families of color. This study represents an important first step to understanding how various observed CHW engagement strategies influence caregiver engagement in BPTs. The engagement strategies CHWs implement naturally as well as those they are trained to deliver can have significant public health implications for underserved communities (35), thus, underscoring the need to evaluate CHW-delivered strategies.

Of the four composites that comprise the ACEs Observational Coding System utilized in the present study as well as the total score, only the alliance composite and the total score demonstrated acceptable internal consistency. This may reflect differences in the nature of the domains assessed. Alliance-based strategies, for example, are relational, which is a cohesive and consistently expressed dimension of engagement (63). However, collaboration, empowerment, and psychoeducation may be more context-dependent strategies based on the needs during a particular session. The present study only captured behaviors occurring during two formal and structured PCIT sessions. That is, data collection may have missed engagement strategies occurring between sessions, such as through informal phone calls involving problem-solving, in which additional collaborative and/or empowerment strategies may have been present. Given the context dependency of these domains, it may be that observation across multiple contexts was necessary for greater generalizability (64). Furthermore, the acceptable internal consistency of the total score may indicate that, when considered all together, the strategies are inclusive of a broader construct of engagement-specific strategies.

Findings indicate CHWs utilize roughly 11 of the 14 original strategies. Overall, strategies were delivered with moderate extensiveness. This is promising, given that a previous study assessing engagement strategies among community-based mental health therapists found low overall use of strategies (27). Interestingly, the original observational tool was designed to evaluate engagement strategies common across evidence-based treatment models (27), showcasing the CHWs’ capacities to utilize the strategies designed for these structured models while maintaining a meaningful relationship with the families they serve. Qualitative studies suggest the culture, community, and experiences CHWs share with the individuals and families they serve equip them with an inherent capacity to relate to and, therefore, engage these communities in treatment (33, 38, 39), which might explain the ways in which CHWs intertwine their natural relatability and the strategies designed for these evidence-based models to promote engagement.

Strategies delivered with high extensiveness (i.e., *M* ≥2) included *CHW provided psychoeducation about child services to caregiver* and *CHW recognized caregiver strengths and effort to participate in therapy*. This was anticipated given the nature of PCIT, which is heavily skills based and involves *in-vivo* coaching (e.g., praising a caregiver for their skill use), in which the skills are reinforced each session and the rationale is provided to promote skill use or desistence (65–67). Though CHWs did not provide *in-vivo* coaching, they were trained to help caregivers practice, review the rationale for, and model the skills, including praise (e.g., praising caregiver's use of skills/strengths in session). Consistent with this training, CHWs frequently used praise as an engagement strategy during sessions, although the present findings do not establish that CHW use of praise contributed to caregivers’ skill acquisition.

This can further be used to understand the finding suggesting that the delivery of psychoeducation is negatively associated with the acquisition of skills. In PCIT, caregivers learn to focus attention on positive child behaviors. Providers supporting families in PCIT model these skills in session. However, it appears as though a focus on child behavioral concerns, even in the context of psychoeducation, can influence overall skill acquisition through unintentional modeling of attention toward behavioral problems. It is important to note the current study only included correlational data and, therefore, conclusions about the direction of and agents driving this relationship cannot be made. Perhaps caregivers of children presenting with more challenging behaviors may be driving these findings by pulling for focus on these challenging behaviors. More detailed exploration of these relationships is important to promoting understanding of how and when CHWs can support families in treatment.

Notably, findings of the current study emphasize the importance of utilizing alliance strategies (i.e., *CHW actively listened to caregiver's perspectives, CHW conveyed caregiver-CHW partnership in child's therapy*, and *CHW communicated positive regard to caregiver*) in the first session to promote engagement among caregivers in treatment. This aligns with evidence from previously conducted studies supporting the importance of first session alliance building between therapists and clients as well as its effect on overall outcomes (68, 69). To the authors’ knowledge, this is the first study to extend the evidence to further support first session alliance building between CHWs and caregivers in treatment.

### 4.1 Limitations

Several relevant CHW strategies—as indicated through previous qualitative studies with CHWs (33, 38, 39)—added to the observational tool utilized in the present study were not included in the analyses conducted due to low base rates (i.e., *CHW leveraged shared characteristics, background, or lived experiences to build trust* and *CHW modeled advocacy to the caregiver when navigating various systems*) or were included, but were delivered with low extensiveness (i.e., *CHW addressed barriers to caregiver participation*). This may be the result of limitations with respect to data collection within the current study, in which only session data were observed and coded. In other words, the current study can only be interpreted when considering the support that CHWs provide with respect to PCIT delivery, which is structured in nature (44, 45). Additionally, only two sessions throughout the course of treatment were coded for engagement strategies. However, the CHWs in the present study also served families through their role at a community-based organization, in which they provided support outside of session (e.g., bridging access to services, addressing barriers, and advocacy (44). Data gathered for the current study, thus, overlooked the possible use of key strategies that could be further promoting engagement among caregivers, which occurred in additional phone calls. Understanding all strategies through which CHWs increase treatment engagement can be critical, given evidence that poor engagement may have broader system-level consequences, included reduced provider self-efficacy and increased provider turnover within community mental health settings (12, 15).

In addition, the current sample size was small and only included Latino caregivers, therefore limiting the statistical power and the generalizability of the current results. The sample of CHWs participating in the delivery of services was also small, further impeding generalizability of engagement strategy utilization observed. The small sample size also restricted psychometric testing of the CHW-modified observational tool used to explore engagement strategies, thus limiting the reliability and validity of the tool used in the present study (70). Moreover, multiple regression models were examined without correction for multiple comparisons, increasing the possibility of Type I error. Accordingly, statistically significant associations should be interpreted cautiously and replicated in larger, adequately powered samples.

### 4.2 Future directions

The findings in the present study demonstrate the ways in which CHWs can provide additional support to underserved families to promote treatment engagement. Given the limitations, future studies should include children and caregivers from various backgrounds to better understand the types of engagement strategies being utilized across diverse communities. In addition, future data collection should include strategies utilized in and out of intervention session to better understand overall engagement support being provided by CHWs as well as future training support needed. Finally, future research should further advance understanding of adaptive use of engagement strategies to optimize support from CHWs in treatment (i.e., for whom and when strategies/CHW-based models are most useful). Given that the CHW engagement strategies observed through the present study differ from what CHWs providing support are trained to do [e.g., addressing barriers, advocacy, leveraging shared experiences; (37)], qualitative methods for exploring these differences should be implemented to achieve a more nuanced understanding of the context of and rationale for the use of these strategies during sessions with families.

### 4.3 Clinical and training implications

The present study also provides information to further training approaches that advance clinical practice among CHWs in the mental health field. The findings suggest alliance-based engagement strategies in the first session are pivotal to engagement throughout treatment among Latino families, leading to increased participation and adherence to mental health interventions. Given that they are already embedded within the communities they serve, CHWs come with natural strategies to engage individuals in treatment that incorporate culturally relevant experiences and perspectives. Future training programs that aim to enhance CHW support among underserved families should focus on methods that adequately prepare CHWs to address mental health and behavioral challenges while augmenting the natural strategies CHWs carry to engage individuals and families in treatment. Deepening the understanding of CHW engagement among families of color is a critical component to promoting more inclusive and accessible environments for individuals seeking and receiving mental health support within these underserved communities.

## Data Availability

The raw data supporting the conclusions of this article will be made available by the authors, without undue reservation.
